# Individual- and population-level effects of childhood adversity and emotional problems on early-onset suicide plans and/or attempt(s)

**DOI:** 10.1186/2049-3258-73-S1-P39

**Published:** 2015-09-17

**Authors:** Philippe Mortier, K Demyttenaere, RP Auerbach, JG Green, RC Kessler, G Kiekens, MK Nock, R Bruffaerts

**Affiliations:** 1 KULeuven, Leuven, Belgium; 2 Research Group Psychiatry, Department of Neurosciences, KU Leuven University, Leuven, Belgium; 3 Harvard Medical School; McLean Hospital, Center for Depression, Anxiety and Stre, Belmont, MA, USA; 4 School of Education, Boston University, Boston, MA, USA; 5 Harvard Medical School, Department of Health Care Policy, Harvard University, Cambridge, MA, USA; 6 Department of Psychology, Harvard University, Cambridge, MA, USA

## Background

Childhood adversity and emotional problems are strong and potentially modifiable predictors for early-onset severe suicidal thoughts and behaviors (STB).

## Objectives

To identify individual-level and population-level risk factors for STB in young people.

## Methods

Web-based selfreport data from incoming KULeuven freshmen (n=4,921; RR=65.4%) were used to calculate multivariate odds ratios (OR), and multivariate population attributable risk proportions (PARP) for the association between lifetime suicide plans and/or attempt(s) on the one, and six types of childhood adversity and 6 types of emotional problems on the other hand.

## Results

Lifetime prevalence (P) of suicide plans and/or attempt(s) was 6.9% (SE=0.3) with an average age of onset of 14.3 years (SE=0.2; SD = 2.5). Multivariate associations were found with frequent victimization of childhood abuse at home (p=3.4%; SE=0.3; OR=3.8; PARP=10.4%), frequent childhood bully victimization (p=10.1%; SE=0.5; OR=2.4; PARP=13.5%), lifetime risk for internalizing disorders (p=37.0%; SE=0.5; OR=6.5; PARP=65.7%), one or more eating disorder symptoms (p=12.0%; SE=0.3; OR=2.6; PARP=15.5%), and one or more psychotic symptoms (p=7.5%; SE=0.3; OR=3.3; PARP=12.0%).

## Limitations

The cross-sectional study design precludes causal inference, and college student findings may not be fully representative for early-onset STB among the general population.

## Conclusion

Early-onset STB is mostly attributable to proximal risk factors such as internalizing mental disorders, eating disorders, and psychotic symptoms. However, distal risk factors like bully victimization and childhood abuse also play a considerable role in the onset of STB among young people. In terms of prevention, our data suggest that resources should preferably be allocated to the early detection of internalizing disorders.

